# Gut-Directed Hypnotherapy May Pose Specific Challenges in Gender-Diverse Patients: A Review of 3 Patients

**DOI:** 10.14309/crj.0000000000001355

**Published:** 2024-05-15

**Authors:** Prianca Tawde, Oakland C. Walters, Jessica K. Salwen-Deremer

**Affiliations:** 1 Department of Internal Medicine, Weill Cornell Medicine at New York Presbyterian, New York, NY, USA; 2 Department of Psychiatry, Te Whatu Ora Te Tai Tokerau, Whangārei, New Zealand; 3 Department of Psychiatry & Center for Digestive Health, Dartmouth Hitchcock Medical Center, Lebanon, NH

**Keywords:** irritable bowel syndrome, disorders of gut-brain interaction, hypnosis, psychogastroenterology, transgender health, gender diversity

## Abstract

Disorders of gut-brain interaction are common and often characterized by chronic symptom courses. While gut-directed hypnotherapy is effective for refractory disorders of gut-brain interaction, the required internal awareness and vulnerability may be challenging. Driven by our own clinical experiences, we conducted qualitative interviews with patients who identified as transgender or gender diverse and who had discontinued gut-directed hypnotherapy. Four main themes were generated from these interviews related to distress resulting from body awareness, difficulty with vulnerability, the importance of gender-affirming supports, and external barriers. Providers are encouraged to consider gender diversity, and more broadly body image, in discussion of hypnosis treatment.

## INTRODUCTION

Over 40% of the population may experience a disorder of gut-brain interaction (DGBI), and gut-directed hypnotherapy (GDH) is an evidence-based intervention for a number of common DGBI.^1-3^ During GDH, patients are asked to be engaged with their bodies, particularly when deepening their relaxation. The intervention also requires patients to be vulnerable with their provider, as the provider guides the hypnosis experience. This combined awareness of the body and vulnerability can be particularly challenging for some patients; for example, GDH is not recommended for individuals with unresolved post-traumatic stress symptoms.^3^ Clinically, our team has seen that patients who are either transgender or gender diverse (ie, their gender identity does not correspond with their sex assigned at birth) report difficulties engaging with GDH. Although there is a growing evidence-based examining engagement in awareness-centered therapies by transgender and gender-diverse individuals, there do not appear to be any published data on hypnotherapy in this population.^4–6^

Understanding the nuances in providing care to transgender and gender-diverse patients is critical, as these individuals are at increased risk of numerous physical and mental health conditions, including DGBI.^7^ In addition, sexual and gender minority individuals are more likely to be stigmatized or dismissed by providers and to avoid care due to stigmatization.^7^ Presently, 1.6% of the US population identifies as transgender or gender diverse, and this percentage is increasing.^8^ Thus, we sought to explore this clinical phenomenon so that we and others can provide better, more informed care.

## CASE REPORT

### Setting

All patients from this case report were receiving care through the Section of Gastroenterology and Hepatology at Dartmouth-Hitchcock Medical Center. In this tertiary care center, GDH is delivered by a cisgender female clinical psychologist using the North Carolina protocol.^9^ A full course of treatment involves 7 hypnosis sessions, and we have adapted this protocol for delivery in a group format.^10,11^ Patients are all referred to this program by their treating gastroenterologist.

Authors conducted qualitative interviews with transgender and gender-diverse patients who had initiated but not completed GDH for a DGBI. A semi-structured interview guide was developed using a trauma-informed care lens, and interviews were conducted using one-on-one video conferencing. Qualitative themes were derived from these interviews. Patients consented to the use of interview data for a published case series, and interviews were determined to be exempt by our institution's Human Research Protection Program (#02001303).

### Cases

We interviewed 3 patients (aged 19–55 years) who were assigned female at birth, reported dysphoria related to their gender identity, and used they/them pronouns. None of the patients completed the full GDH intervention at least partially due to intervention-related discomfort. Patient-specific information is as follows.

1. Patient A: Identified as transgender and nonbinary. Their pertinent medical history included anxiety and irritable bowel syndrome. They completed 2 sessions of virtual group-based GDH before dropping out of the program. They were offered to instead complete one-on-one sessions, and declined, preferring instead to continue individual work with their community therapist. They indicated that prior individual attempts at mind-body work had also been anxiety provoking and unsuccessful.
2. Patient B: Identified as nonbinary and androgynous, describing their gender as “a work in progress.” Their pertinent medical history included anxiety and globus sensation. The patient tried one-on-one GDH once and attempted group relaxation training twice (session 1: diaphragmatic breathing; session 2: mindfulness). Neither of these treatments were tolerated, and individual therapy sessions pivoted to cognitive behavioral therapy.
3. Patient C: Identified as nonbinary. Their pertinent medical history included anxiety, depression, pelvic floor dysfunction, and Ehlers Danlos-related intestinal dysmotility. They completed 2 sessions of virtual, group GDH before dropping out of the program, indicating that they preferring to incorporate techniques into pelvic floor physical therapy as that intervention was logistically easier.

### Themes

The broad themes that were identified across interviews were (i) awareness of the body can be distressing, (ii) vulnerability required for hypnotherapy can be difficult, (iii) creating a gender-affirming environment can reduce healthcare-related stressors, and (iv) external barriers can contribute to disengagement with hypnotherapy. In addition, patients each contributed valuable and unique perspectives about their experiences.

*Awareness of the body can be distressing*. During hypnotherapy, patients are guided through engaging with and relaxing parts of their body. Patient A specifically highlighted discomfort with this experience, citing years of battling with body image related to gender dysphoria. Being asked to focus on their body was anxiety inducing rather than relaxing. Patient B highlighted how self-perception and body image had been tied to discomfort in their day-to-day life.

*From the get-go, it was deeply stressful. I think I am a person that I am mostly deeply uncomfortable with my own body…. To live safely I do not think about [my body]. This practice had me thrown into the deep end. ‘let's sit and be internal.’ Did not feel good for me. Just learning to run when I can barely walk (Patient A)*

*Vulnerability required for hypnotherapy can be difficult and is amplified by having to disclose information to an unknown group.* Patients feared the perceptions of others and the unknowns of group care, though noted that the virtual format felt safer than in person. Even patient C, who felt comfortable in the group setting, recognized the difficult nature of vulnerability in a patient group for people experiencing gender dysphoria.

*That people are perceived by other people is mortifying. Again, these people don't know me. It's a minefield. You are vulnerable and not prepared to do that (Patient A)*

*I can tell people [I am non-binary], but part of why I haven't committed to transition is that I can hide in plain sight. Safety is a real concern (Patient B)*

*Gender-affirming supports and community can reduce healthcare-related stress.* Patient C described how a history of therapy from a young age and a supportive community at university allowed them to be comfortable in their identity and body image, while patient B shared how trust in an institution helped them open up in therapy.

*I did therapy for a while when I was younger, and it is not uncommon to step into myself and connect with myself… It is uncomfortable to have a differing identity from the way you appear (Patient C)*

It was refreshing *to have someone talk to me about my gender… I had faith in her because she was affiliated with Dartmouth (Patient B)*

*Barriers to hypnotherapy.* All 3 patients cited other reasons beyond discomfort for discontinuing treatment, including preference for incorporating techniques into preexisting therapeutic relationships, distrust in the healthcare system, and scheduling difficulties. Broadly, patients suggested including more time to prepare for the intervention into the group curriculum, ability to turn off one's camera, and collaboration between the gastrointestinal (GI) psychologist and outside treatment providers as ways to improve their experience. One patient reported continuing treatment with a GI psychologist but discontinued the use of hypnotherapy as an intervention. Another elected to continue use of hypnotherapy in the context of individual visits with a pelvic floor physical therapist.

## DISCUSSION

Given current recommendations to use brain-gut behavior therapies, including hypnosis, in the care of patients with DGBI, it is critical that we understand the limitations of these interventions and/or methods of delivery. As providers, we need to be aware of how to better care for the growing number of patients who experience dysphoria related to gender. Although this case report provides an introduction to possible treatment considerations for transgender or gender-diverse patients, it is not without limitations. First, we only interviewed transgender and gender-diverse patients who discontinued this intervention, and not those who completed it, subjecting our findings to possible bias. We are not aware of any transgender or gender-diverse patients who did complete the intervention, although medical record-based identification of gender may not be fully accurate. In addition, patients interviewed reported concerns related both to the therapy itself and to the group format. Vulnerability in a group setting and logistical barriers are not unique to this population, and while group treatment may improve access, it also may not be appropriate for some patients.^10,12^ In our own program, we have made a number of adaptations to the delivery of group GDH to address some of the concerns described above, though these adaptations may not be enough for some patients.^11^

A 2022 Rome Working Team Report suggested that patients with more complex mental health needs may not be appropriate for brain-gut behavior therapies.^3^ Indeed, recent research suggests that patients with higher baseline depression are less likely to experience improvements in pain with GDH, and patients with greater anxiety and/or lower quality of life at baseline are more likely to drop out.^13^ Given the high rates of both discrimination and mental health concerns in individuals who identify as transgender or gender diverse, it is likely that for these patients, shared decision making is particularly important.^14^ It is possible that patients who self-identify as transgender or gender diverse and who are actively experiencing gender dysphoria may have better outcomes with individual therapy, including engaging in other evidence-based brain-gut behavior therapies or working first with a therapist who specializes in gender-related concerns.^3^

The case of patient B exemplifies this process. Following their adverse reaction to both relaxation therapy and GDH, they transitioned to a cognitive therapy approach, including working with the psychologist to explore, the reasons behind the adverse reaction. Through this process, patient B was able to recognize that their lifelong concerns related to body image, sexual orientation, and eating behaviors were in part related to an underlying noncisgender gender identity. They began working with a therapist specializing in (trans)gender-related issues alongside the GI psychologist, eventually reducing some of their GI symptoms and terminating GI-specific treatment, while continuing to address concerns related to gender identity.

Broadly, these cases highlight challenges providers should be aware of when delivering GDH to patients who identify as transgender or gender diverse, particularly when gender dysphoria is present. However, the experiences of a few individuals cannot speak for the transgender and gender-diverse population as a whole. As a medical community, we should continue to work toward both better understanding the specific needs of these patients and fostering gender-affirming spaces.

## DISCLOSURES

Author contributions: P. Tawde designed this study with critical input from JK Salwen-Deremer. P. Tawde was responsible for acquisition of data under JK Salwen-Deremer's supervision and P. Tawde, OC Walters, and JK Salwen-Deremer interpreted the data. P. Tawde developed the initial draft of the manuscript and OC Walters and JK Salwen-Deremer revised it critically for important intellectual content. All authors have approved this version of the manuscript and agree to be accountable for accuracy and integrity of the work. JK Salwen-Deremer is the guarantor of this manuscript.

Financial disclosure: This work was supported by the Susan and Richard Levy Health Care Delivery Incubator, a joint venture between the Dartmouth Institute for Health Policy & Clinical Practice and Dartmouth Health (JK Salwen-Deremer).

Patients consented to the use of interview data for a published case series, and interviews were determined to be exempt by our institution's Human Research Protection Program (#02001303).
